# Polypyrrole with Embedded Carbide-Derived Carbon with and without Phosphor Tungsten Acid: Linear Actuation and Energy Storage

**DOI:** 10.3390/polym14214757

**Published:** 2022-11-06

**Authors:** Zane Zondaka, Quoc Bao Le, Rudolf Kiefer

**Affiliations:** 1 Intelligent Materials and Systems Lab, Institute of Technology, University of Tartu, Nooruse 1, 50411 Tartu, Estonia; 2 Conducting Polymers in Composites and Applications Research Group, Faculty of Applied Sciences, Ton Duc Thang University, Ho Chi Minh City 700000, Vietnam

**Keywords:** PPyCDC, PPyCDC-PT, linear actuation, energy storage, organic and aqueous electrolytes

## Abstract

Researchers have focused on incorporating porous carbon particles such as carbon-derived carbon (CDC) into polypyrrole (PPy), preferably on the surface, to achieve high-capacitive electrodes. Less attention is afforded to their linear actuation properties. Therefore, in this work, we chose two different electropolymerization processes using the typical PPy doped with dodecylbenzene sulfonate (DBS^−^) and added CDC particles, compared with CDC with phosphotungstic acid (PTA), forming CDC-PT^4−^ dopants. The resulting PPy/DBS-CDC (PPyCDC) and PPy/DBS-CDC-PT (PPyCDC-PT) films showed different morphologies, with PPyCDC having the most CDC particles on the surface with less surrounding PPy, while in PPyCDC-PT, all the CDC particles were covered with PPy. Their linear actuation properties, applying electrochemical techniques (cyclic voltammetry and square wave potential steps), were found to enhance the PPyCDC-PT films in organic (2-times-higher strain) and aqueous electrolytes (2.8-times-higher strain) in an applied potential range of 0.8 V to −0.5 V. The energy storage capability found for the PPyCDC was favorable, with 159 ± 13 F cm^−3^ (1.2 times lower for PPyCDC-PT) in the organic electrolyte, while in the aqueous electrolyte, a result of 135 ± 11 F cm^−3^ was determined (1.8 times lower for PPyCDC-PT). The results showed that PPyCDC was more favorable in terms of energy storage, while PPyCDC-PT was suitable for linear actuator applications. The characterization of both the film samples included scanning electron microscopy (SEM), Raman, FTIR, and energy-dispersive X-ray (EDX) spectroscopy.

## 1. Introduction

The trend in society demands electronic devices operated at a low voltage that can store energy. Carbon materials (electric double-layer capacitors, non-faradaic process [1]) are poor conductors, while pseudo-capacitors [2] such as polypyrrole (redox reactive, faradaic process) are conductive in the charged state. In most cases, these are combined [3], forming hybrid materials with an enhanced capacitance and with promising results [4] for a new generation of supercapacitor devices. Focusing on specific applications, such as smart textiles, materials with actuation properties for conducting polymers have recently been described [5]. Dual functionality with added energy storage in the case of nanomaterials always opens up new applications suitable for healthcare [6], such as electronic skin [7] or smart patches. Mesoporous carbon materials [8] used as hollow tubes [9] for potassium ion storage devices [10,11,12] and batteries [13] have been investigated. Several carbon-related materials have been introduced in conducting polymers. Carbide-derived carbon materials [14] show a high specific capacitance [15] due to their narrow pore size under 1 nm. 

The incorporation of these hydrophobic CDC materials into PPy polymerized in an aqueous solution can be achieved in two ways. Firstly, one can achieve this by using NaDBS as an electrolyte, where micelles form around the pyrrole and CDC materials, polymerizing them together and creating Ppy/DBS-CDC (PPyCDC) composites. The other way was introduced in [16], using PTA additives forming multi-charged polyanions with CDC particles (CDC-PT^4−^) with composites contained in PPy/DBS-CDC-PT (PPyCDC-PT) films. Recent research [17,18] revealed that the addition of PTA to PPy forms an asymmetric capacitor providing a high specific capacitance. 

Our goal in this work was to investigate these two different film samples, PPyCDC and PPyCDC-PT, in terms of their linear actuation properties in lithium bis(trifluoromethanesulfonyl)imide (LiTFSI) in aqueous (aq) and propylene carbonate (PC) solvents, combined with a determination of their volumetric capacitance.

The electropolymerization of PPy doped with DBS^-^ takes place in an aqueous monomer solution, where, at the applied potential, the pyrrole is oxidized, forming radical cations. Combined with dimer and longer oligomers, those became insoluble and are deposited on the working electrode [19]. The lower the deposition process is, the denser and more compact the films become, ideally being created at low temperatures (here at −20 °C, with the addition of anti-freezing agents such as ethylene glycol). 

The principal actuation mechanism of PPy/DBS in aqueous electrolytes with CDC or CDC-PT is related to solvated cation ingress (cation-driven actuator) at the point of reduction due to the immobile DBS^-^ anions and charged CDC-PT^4−^ macro-anions (upon oxidation, these balance the positive mobile charge), leading to expansion (volume change). In the case of those materials actuated in an organic solvent, expansion at oxidation has mainly been found for PPy/DBS [20]. The reason for this is that those DBS^-^ cation^+^ pairs, at reduction, cannot dissociate in organic solvents [21] (the same was found for PPyCDC-PT samples [22]), with a new place occupied at oxidation, causing the former cation-driven actuator to be anion-driven. 

Cyclic voltammetry and square wave potential steps (frequency range of 0.0025 Hz to 0.1 Hz) of the PPyCDC and PPyCDC-PT films were performed in combination with linear actuation investigations of the LiTFSI-aq and LiTFSI-PC electrolytes at an applied potential range of 0.8 V to −0.5 V. The volumetric capacitance was determined based on chronopotentiometry measurements. The film samples’ characterization included SEM imaging, the electronic surface conductivity, and the ion content, as well as element determination at oxidation/reduction after the actuation cycles, evaluated using EDX spectroscopy. The material characterization aiming to identify PTA and CDC in the film samples was performed using FTIR and Raman spectroscopy.

## 2. Material and Methods

### 2.1. Chemicals

Amorphous-titanium-carbide-derived carbon (TiC-800, BET surface area 1470 m^2^g^−1^, average particle size 1–3 μm, micropore volume 0.57 cm^3^g^−1^, average pore size 1.02 nm) was purchased from Skeleton Technologies Ltd. (Tallinn, Estonia). The solvents, including propylene carbonate (PC, 99%), ethylene glycol (EG, 99.8%), and ethanol (technical grade), were purchased from Sigma-Aldrich (Taufkirchen, Germany). The pyrrole (Py, ≥98%, Sigma Aldrich) was vacuum distilled before use and stored at a low temperature. Lithium bis(trifluoromethanesulfonyl)imide (LiTFSi, 99.95%) was obtained from Solvionic (Toulouse, France). Sodium dodecylbenzenesulfonate (NaDBS, technical grade) and polyoxometalate (POM) in the form of phosphotungstic acid (PTA, PW_12_O_40_^3−^) hydrate were purchased from Sigma-Aldrich and used as supplied. Milli-Q+ (Tallinn, Estonia) water was applied for the solutions. 

### 2.2. Electropolymerization

The electrochemical synthesis of the electroactive PPy films was carried out in a two-electrode electrochemical cell that contained a stainless steel sheet (18 cm^2^) as the working electrode and stainless steel mesh as the counter electrode. The bath contained 0.1 M NaDBS in EG:H_2_O (50:50 wt.%), 0.1 M pyrrole, 1% CDC, and 0.1 M phosphotungstic acid (PTA), forming PPy/DBS-CDC-PT (PPyCDC-PT) films. The same conditions were applied to obtain the PPy/DBS-CDC films, with no PTA inclusion. Both solutions were sonicated for 30 min in an ice bath and stored in the fridge before the electrochemical polymerization. Galvanostatic electropolymerization was carried out at 0.1 mA cm^−2^ for 40,000 s (11.1 h) at −20 °C. After polymerization, the films were extracted from the stainless steel and washed in ethanol to remove the residues of pyrrole and then in Milli-Q+ to remove the excess NaDBS and PTA. The films were dried in an oven at 60 degrees (2 mbar) for 12 h. The thickness of the PPyCDC-PT films was found to be in the range of 19 ± 0.6 µm, while PPyCDC had a thickness of 18 ± 0.5 µm. The films were stored in electrolytes 24 h before the measurements, with the thickness determined using an electronic micrometer gauge meter (Dainu, 0.001 mm sensitivity), showing a thickness of 21 ± 1.5 μm for PPyCDC and PPyCDC-PT, while more swelling was observed in the LiTFSI-aq electrolyte with both films, which had a comparable thickness of 25 ± 2 μm.

### 2.3. Linear Actuation Measurements

The PPyCDC and PPyCDC-PT films were cut in dimensions of 1.0 cm × 0.1 cm. After applying the films, the working electrode was fixed on the lower holder with gold contacts and an upper clamp connected to the force sensor (TRI202PAD, Panlab, Barcelona, Spain) of the homemade ECMD device [23,24]. The fixed films (working electrode) were placed in a three-electrode cell with a platinum sheet as the counter electrode and an Ag/AgCl (3 M KCl) reference electrode in either an aqueous or propylene carbonate (0.2 M LiTFSI) electrolyte. The films were stretched in the range of 1% for 8 h before the measurements in the electrolyte. The process was performed to ensure that the swelling of the films in the applied solvents did not influence the linear actuation measurements. To evaluate the elastic modulus of the films before and after actuation, we employed the ECMD device’s movable linear actuation stage, where the factor k (mg/μm) was determined before and after the actuation cycles.

Linear actuation measurements (constant force of 4.4 mN with a free length between both clamps of 2 mm) are obtained in real-time using software [23] that connects the linear actuation parameter with the signals of the potentiostat (Biologic PG581, Seyssinet-Pariset, France). The strain ε in % was obtained from the formula ε = ΔL/L∙100% (with ΔL = L − L_1_, L free length of PPy films and L_1_ the length change at actuation). Cyclic voltametric measurements (scan rate: 5 mV s^−1^), square wave potential steps (frequency range: 0.0025 Hz to 0.1 Hz), and chronopotentiometry (current steps from ±0.05 mA to ±2 mA having a constant charge of ±10 mC) in the applied potential range of 0.8 V to −0.5 V were performed. 

### 2.4. Characterization

Both the PPyCDC and PPyCDC-PT films were characterized directly after polymerization using scanning electron microscopy (SEM, VEGA Tescan, TESCAN ORSAY HOLDING, Brno-Kohoutovice, Czech Republic). Energy-dispersive X-ray (EDX) spectroscopy (Oxford Instruments with an X-Max 50 mm^2^ detector, High Wycombe, UK) of the film samples was performed after the actuation cycles on cross-section area that was oxidized (+0.8 V for 2 min) and reduced (−0.5 V for 2 min). Fourier transform infrared spectroscopy (FTIR, 1600–500 cm^−1^) measurements were performed using a Bruker Alpha spectrometer (Bruker Alpha, with Platinum ATR, Billerica, MA, USA). Raman spectroscopy (2000 cm^−1^–500 cm^−1^) of the film samples and pristine CDC were conducted with a 632.8 nm laser using a micro-Raman spectrometer (Renishaw, Wotton-Under-Edge, UK). The electronic surface conductivity of the dried samples was determined by a four-point probe method using a surface resistivity meter (Jandle 4-Point Probe Head, Model RM2, Leighton, Buzzard, UK).

## 3. Results and Discussion

The addition of CDC materials to PPy doped with DBS^−^ films can be performed either by adding PTA [16] to place these CDC particles over the added charges in an aqueous monomer solution or by using the dopant DBS^−^ to solubilize the hydrophobic CDC particles [25] on the micelles. Thus far, a comparison of both types of PPyCDC and PPyCDC-PT films has not been performed; thus, the aim of this work was to analyze the linear actuation properties of the materials in organic and aqueous electrolytes and their energy storage capability. CDC particles are known to be microporous materials [14] that are mainly applied in supercapacitor applications but have also been shown to have actuation properties [26]. At the same time, the mechanism referred to as the non-faradaic process is based on forming an electrical double layer at the applied voltage on the CDC surfaces. Previous research [22] showed that PPyCDC-PT films mainly undergo faradaic processes, with the CDC particles surrounded by PPy chains [27]. 

### 3.1. Characterizations of the PPyCDC and PPyCDC-PT Films

Several characterizations of the PPyCDC and PPyCDC-PT films, such as SEM imaging, were conducted to study the differences occurring with or without PTA addition. The main questions are as follows: if no PTA is applied during electropolymerization, are the CDC particles covered with PPy, and how are they distributed in the PPyCDC films? The film compositions were analyzed with FTIR and Raman spectroscopy to determine if the PTA included the CDC particles can be detected. The qualitative EDX spectroscopy of a cross-section image was performed on the film samples after the actuation cycles to evaluate which elements could be detected at oxidation (0.8 V) and reduction (−0.5 V).

#### 3.1.1. Electropolymerization, SEM Images, and Electronic Conductivity

**Figure 1:**
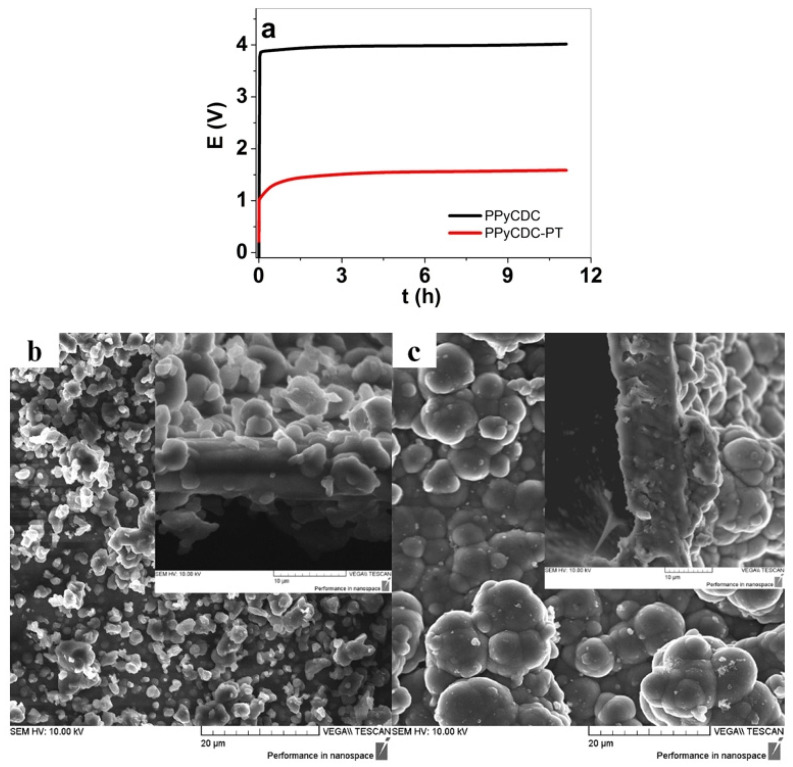
(**a**) Galvanostatic electropolymerization (0.1 mA cm^−2^, 11.1 h, −20 °C, EG:Milli-Q+ 50:50 wt.%) in a two-electrode cell forming PPyCDC (black curve) and ppyCDC-PT (red curve). The SEM images of the surface (scale bar 20 μm), with the inset of cross-section image (scale bar 10 μm), showing (**b**) PPyCDC and (**c**) PPyCDC-PT films.

The electropolymerization of PPyCDC and PPyCDC-PT are presented in Figure 1a. The PPyCDC and PPyCDC-PT films were characterized using surface and cross-section SEM images, as shown in Figure 1b,c.

It is well known that PTA, functionalized as a catalyst in electropolymerization [28], can be observed in the much lower potential evolution of PPyCDC-PT. Figure 1a demonstrates this at the end of the polymerization at 1.58 V, while for PPyCDC, 4.05 V was detected. In galvanostatic polymerization, the lower the potential is, the more dense and compact the film becomes, and the conductivity increases. The general electropolymerization of PPy using DBS^-^ as a dopant leads to the pyrrole’s micelle formation. It is well known that these micelles of the bulky amphiphilic DBS^−^ anions surrounding the hydrophobic particles, either pyrrole [29] or CDC [25], are formed in an aqueous solution. The electropolymerization of pyrrole around those micelles with chains of PPy of a sufficient length became insoluble [19] and are deposited on the working electrode. The larger hydrophobic CDC particles form larger negatively charged micelles. We assume that those micelles differing in their size, which lower the ion conductivity of the monomer solution, are the main reason why the electropolymerization required such a high voltage to form PPyCDC films. The SEM surface and cross-section image shown in Figure 1b revealed that most of the CDC particles decorated the surfaces and were partly covered with PPy, with only a few shown in the cross-section image. 

In the case of PPyCDC-PT formation, the CDC particles are solubilized over PTA, working as polyanions, as shown in the case of other carbon materials [30], wrapping around those leading to a better suspension in aqueous solutions. The CDC-PT^4−^ are an additional dopant added to PTA molecules, as mediators in electropolymerization, forming PPyCDC-PT composites, with most of the CDC particles surrounded by PPy [16], as shown in the SEM images in Figure 1c. The typical cauliflower structure [31] can be observed, with all the CDC particles combined with the PPy. The surface conductivity of the PPyCDC and PPyCDC-PT films, combined with the elastic modulus determination from the stiffness measurements (factor k) before and after actuation, are compared in light of the different applied solvents, PC and aq in the LiTFSI electrolyte, with the results presented in Table 1.

The pristine PPyCDC electronic conductivity was comparable to that of PPy/DBS [32]. It decreased after actuation in LiTFSI-aq and LiTFSI-PC. The PPyCDC-PT film had a 1.3-times-higher conductivity than PPyCDC, with a similar decrease after the aqueous or PC solvent actuation cycles. The elastic modulus showed a slight increase in LiTFSI-aq after actuation. At the same time, PPyCDC-PT revealed a 2.1-times-lower value after the actuation cycles, with similar findings observed in former research [16]. A similar tendency is also found for the PPy/DBS-PT films, with the addition of PTA leading to a decrease in the elastic modulus after the actuation cycles [18]. In LiTFSI-PC, the elastic modulus increased nearly 2.8 times for PPyCDC and 1.2 times for PPyCDC-PT, which was also observed in recent research [22].

#### 3.1.2. FTIR and Raman Spectroscopy

**Figure 2:**
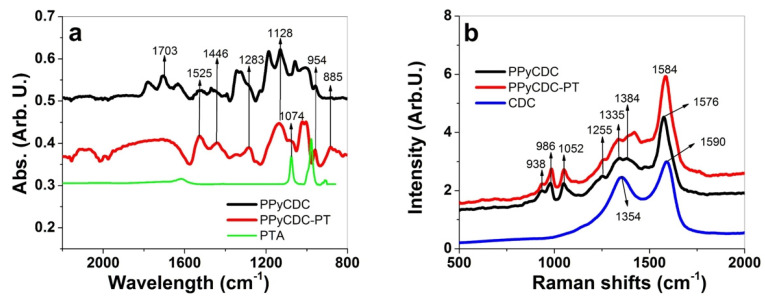
FTIR spectroscopy (2300 cm^−1^–800 cm^−1^) of PPyCDC (black line), PPyCDC-PT (red line), and PTA (green line) are shown in (**a**). Raman spectroscopy (632.8 nm laser) between 2000 cm^−1^–500 cm^−1^ for PPyCDC (black line), PPyCDC-PT (red line), and pristine CDC (blue line) are displayed in (**b**).

To analyze the PPyCDC and PPyCDC-PT films, FTIR spectroscopy was performed, with the results shown in Figure 2a. The Raman spectroscopy findings, with the included pristine CDC, are presented in Figure 2b. 

The typical PPy signals in FTIR spectroscopy [33,34,35] (Figure 2a) are shown at 1525 cm^−1^ and 1444 cm^−1,^ which are attributed to to C=C and C-C PPy ring stretching vibrations. The 1283 cm^−1^ peak represents C-N stretching vibrations, and the 1128 cm^−1^ vibration represents C-N trying vibrations [36]. The peak at 956 cm^−1^ represents C-C out of plane deformation [37], and the 885 cm^−1^ band shows the doping state of PPy, which is much more pronounced in PPyCDC-PT. The main reason for the higher doping state of PPy is reflected in the addition of PTA at polymerization (CDC-PT^4−^), which functionalized as a light oxidant. Another broad peak above 2000 cm^−1^, which is found at 2121–2065 cm^−1^, was described as if a more substantial peak appears, as shown for PPyCDC-PT, for which higher doping levels were indicated [34]. The appearance of the 1703 cm^−1^ peak (C=O vibration) for PPyCDC indicated over-oxidation [38], as expected, in the electropolymerization curve in Figure 1a, in the context of high voltage acceleration in the aqueous monomer solution. The specific PTA lines at 979 cm^−1^ belong to the stretching mode of the terminal. W-O groups could not be identified in PPyCDC-PT, while a peak at 1074 cm^−1^ that describes the stretching vibration of P-O bonds [30] was detected.

Raman spectroscopy (Figure 2b) of the PPyCDC found the dominant peak (C=C backbone stretching [36]) at 1576 cm^−1^, which was shifted in the case of PPyCDC-PT to 1584 cm^−1^ and was shown in recent work as being the reason for the higher oxidation state [39]. The incorporation of PTA and CDC-PT into PPy, as shown in the previous research [27], leads to higher doping states, which were shown here as well, in comparison to PPyCDC. Additional peaks at 930 cm^−1^ and 986 cm^−1^ show the ring deformation bands accelerated to dipolar (di-cation) and polaron (radical cation) states [40], respectively. The 1052 cm^−1^ peak represents the C-H in-plane deformation, and the 1255 cm^−1^ band belongs to the N-H in-plane deformation. Another pair of double peaks shown at 1335 cm^−1^ and 1384 cm^−1^ belong to the ring stretching mode of PPy. A more substantial peak at 1384 cm^−1^ indicates a higher oxidation state of PPy [40], which is enhanced in PPyCDC-PT. Pristine CDC has two peaks, one shown at 1354 cm^−1^, which belongs to the D-band, and the 1590 cm^−1^ peak, which shows the G-band [41]. In general, the PPy bands cover all the CDC intensities. At the same time, a more pronounced shape of the D-band in the range of 1354 cm^−1^–1384 cm^−1^ can be detected for PPyCDC, where most CDC particles are found on the surface of the films (SEM images shown in Figure 1b). The most dominant PTA band found at 1000 cm^−1^ indicates the W=O stretch [42], which was not detected due to the overlapping PPy bands of PPyCDC-PT in this wavelength range. 

In summary, as shown by FTIR spectroscopy, the identification of PTA was discovered for the PPyCDC-PT samples. In contrast, in the Raman spectroscopy analysis, the shifts and intensities of certain bands showed that PPyCDC-PT has a higher oxidation state than PPyCDC. The shape of the CDC D-band was more prominent in PPyCDC than in PPyCDC-PT. A further analysis of EDX spectroscopy was performed to quantify the element contents produced and which ions could be detected at oxidation and reduction.

#### 3.1.3. EDX Spectroscopy

**Figure 3:**
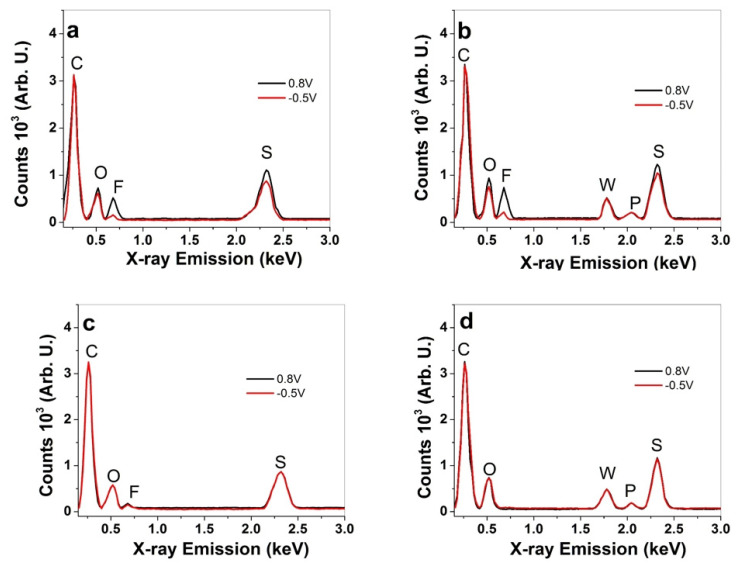
EDX spectroscopy of the counts against X-ray emission at oxidation (0.8 V, black line) and reduction (−0.5 V, red line) performed after the actuation cycles: cross-section image in LiTFSI-PC showing the (**a**) PPyCDC and (**b**) PPyCDC-PT samples. The spectra in the LiTFSI-aq electrolyte are presented for (**c**) PPyCDC and (**d**) PPyCDC-PT.

The elemental composition of the film samples on the cross-section image after the actuation cycles was assessed using EDX spectroscopy at oxidation (0.8 V) and reduction (−0.5 V). The results for the PPyCDC and PPyCDC-PT films in the LiTFSI-PC electrolyte are shown in Figure 3a,b, and those in LiTFSI-aq are displayed in Figure 3c,d.

The typical signals in the EDX spectroscopy of the PPyCDC samples (Figure 3a,c) are shown at 0.26 keV for carbon (C), 0.52 keV for oxygen (O), 0.68 keV for fluoride (F), and 2.32 keV for sulfur (S). The inclusion of PTA forming the PPyCDC-PT films (Figure 3b,d) reveals additional signals that are attributed to PT^4−^ anions at 1.78 keV for tungsten (W) and 2.04 keV for phosphor (P). These do not change their counts in the oxidation and reduction states, showing that those phosphotungstic anions, either as separated anions or anions attached to CDC, do not alter in the redox reaction, as has also been shown in recent research [22,43]. The immobile DBS^-^ anions in the PPyCDC and PPyCDC-PT films are identified by the sulfur and oxygen peaks. The TFSI^-^ anions have fluoride, sulfur, and oxygen contents. Due to their small size, the Li^+^ cations cannot be detected by EDX spectroscopy. Comparing the EDX spectra of PPyCDC and PPyCDC-PT (LiTFSI-PC) in Figure 3a,b, we see that the sulfur, fluoride, and oxygen are reduced at reduction. Therefore, TFSI^−^ anions move in at oxidation, with a small amount still being found at reduction. In the LiTFSI-aq electrolyte, in the case of PPyCDC, shown in Figure 3c, the sulfur peak and oxygen peak do not change significantly at oxidation or reduction.

In contrast, a small fluoride peak was detected at oxidation, with minor changes at a reduction. In the case of PPyCDC-PT, the sulfur and oxygen peaks and the tungsten and phosphor peaks do not alter from oxidation to reduction, which can be attributed to immobile DBS^−^ and PT4^−^ anions, respectively. Therefore, it is reported from previous research [16] that cations Li^+^ are mainly incorporated at reduction. 

### 3.2. Linear Actuation

PPy doped with DBS^-^ is one of the most widely studied conducting polymer actuators. Due to the immobile DBS^-^, the actuation takes place through cations, with solvent incorporation upon discharge to balance the DBS^-^ anions. The PPy/DBS is named a cation-driven actuator. Recent research discovered that, if the solvent changes from aqueous to propylene carbonate, the actuation direction changes, causing the cation-driven actuator to become anion-driven [21]. The main reason for this phenomenon relies on the incapability of the immobile DBS^−^ Li^+^ to dissociate, with the consequence that, in the solvent propylene carbonate, new places are occupied by TFSI^-^ anions, and expansion at oxidation takes place [44]. The incorporation of CDC-PT^4−^ into PPy has also been shown to be affected by solvent propylene carbonate, with the main expansion of the PPyCDC-PT films occurring at oxidation [22]. This work investigates the linear actuation properties of PPyCDC and PPyCDC-PT films, including the solvent change from aqueous to propylene carbonate. In the case of PPyCDC, we observed from the analytics that CDC is mostly shown on the surface of the films, with some CDC particles. Those particles do not contain PPy, while in the case of the PPyCDC-PT samples, all the CDC particles are covered with PPy. Therefore, we aimed to investigate whether CDC has a non-faradaic actuation mechanism and PPy faradaic processes, and whether any effects might influence the linear actuation. Different electrochemical techniques, including cyclic voltammetry and chronoamperometry (frequencies 0.0025 Hz to 0.1 Hz), were used to evaluate the differences between these two samples. For each PPyCDC and PPyCDC-PT film in each of the applied solvents, at least three samples, independent of one another, were formed during electropolymerization. Their linear actuation properties were determined, with the results given as mean values and standard deviations.

#### 3.2.1. Cyclic Voltammetry

**Figure 4:**
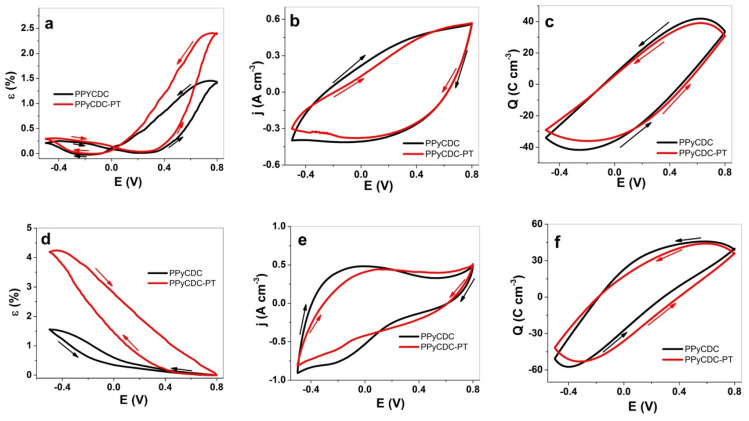
Cyclic voltammetry (scan rate 5 mV s^−1^) of PPyCDC (black line) and PPyCDC-PT (red line) films investigated in LiTFSI-PC electrolytes, showing (**a**) strain ε, (**b**) current density j, and (**c**) charge density Q against potential E (0.8 V to −0.5 V). The films used in LiTFSI-aq are presented with (**d**) strain, (**e**) current density j, and (**f**) charge density Q in the same potential range E. The arrows represent the start and end of the 3rd cycle.

The strain, current density, and charge density curves in the applied potential range of 0.8 V to −0.5 V in LiTFSI-PC for PPyCDC and PPyCDC-PT are presented in Figure 4a–c, with those from the investigation of the LiTFSI-aq electrolyte shown in Figure 4d–f. 

The strain curve for LiTFSI-PC, presented in Figure 4a, revealed the main expansion at oxidation for both the PPyCDC and PPyCDC-PT films. The differences are shown in the PPyCDC-PT that has a 1.7-times-higher strain at oxidation (2.4%) than PPyCDC (1.4%), with a small extension at reduction in the 0.2–0.27% strain range. The main reason for the difference in the strain response of PPyCDC-PT can be drawn back to the change in the elastic modulus (Table 1), which was less intense than that of PPyCDC (1.4 times higher) when actuated in LiTFSI-PC.

The current density curves in Figure 4b revealed no oxidation or reduction peaks with more capacitive shapes [22]. The charge density curves shown in Figure 4c, having close loops, showed that the charging/discharging are in balance [45]. A slightly higher charge density was found for PPyCDC, with 69 C cm^−3^, and PPyCDC-PT, having 63 C cm^−3^. The film samples applied to the electrolyte LiTFSI-aq, in regard to the strain (Figure 4d), showed the main expansion at reduction, as expected for PPyCDC, with the strain found to be enhanced by 2.6 times for PPyCDC-PT (4.2%) compared to PPyCDC, with 1.6% strain. Compared with PPyCDC, where most of the CDC is located on the polymer surface, which will increase the elastic modulus of the film, when CDC was combined with PTA (CDC-PT^4−^), most of those particles in the PPy network were included on the surface, covered with PPy, directly impacting the elastic modulus of the films (Table 1) and affecting the strain [16]. Oxidation peaks for PPyCDC in LiTFSI-aq (Figure 4e) were found at −0.1 V, comparable to the PPy/DBS samples [32], with the shift to 0.1 V for PPyCDC-PT shown to be the reason for PT^4−^’s possession of antioxidant properties [46]. The reduction peaks for both the films were found at −0.2 V. Surprisingly, the charge density (also reflected in the close loops in Figure 4f) of PPyCDC was found to be enhanced by 1.1 times, at 90.4 C cm^−3^, compared to PPyCDC-PT (81.2 C cm^−3^). One reason for such an increasing charge density relates to the microporous CDC particles, as seen from the SEM images in Figure 1b, which are partly uncovered by PPy. Therefore, in LiTFSI-PC and more prominently in the LiTFSI-aq electrolyte, the charge densities of the PPyCDC films increased but did not affect the electromechanical properties. In general, CDC-based electrodes, following EDL formation, showed expansion at discharge either linearly or in bending devices [26]. To obtain a specific expansion of CDC materials obtained through EDL-formation-induced charge injection [1], as shown in previous research [47], the thickness of the linear CDC films was 160 μm, most often combined with a PVdF-HFP binder having a maximum strain of 0.5% in an aqueous electrolyte. Therefore, if we compare such linear CDC films with the PPyCDC sample, the CDC on PPy is randomly distributed, with a contribution to the charging but no effect on the linear expansion. Further linear actuation in square wave potential steps is described in the next section.

#### 3.2.2. Square Wave Potential Steps

**Figure 5:**
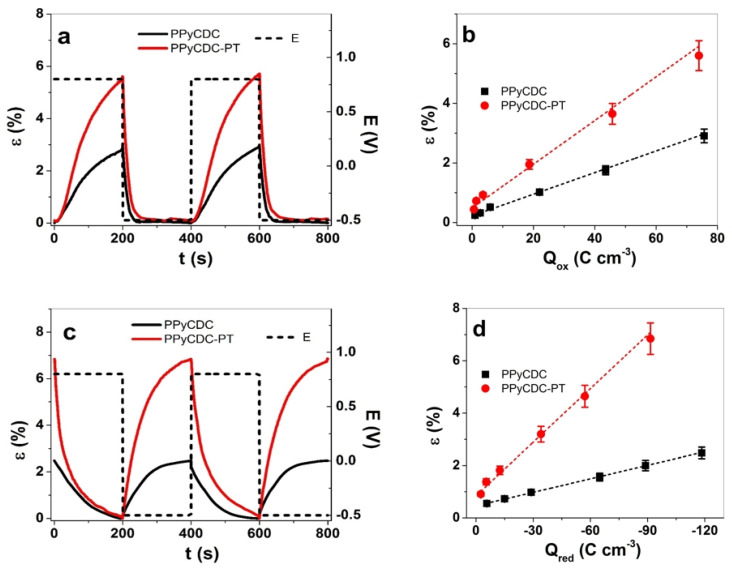
Square wave potential steps of PPyCDC (black line, ■) and PPyCDC-PT (red line, ●) showing strain–time profile at 0.0025 Hz in the potential range E (0.8 V to −0.5 V, black dashed line), following the 3rd to 4th cycle in (**a**) LiTFSi-PC, and (**b**) the strain against the charge densities at oxidation. In the electrolyte LiTFSI-aq, the strain–time profiles of both film samples are shown in (**c**), and the strain against charge density at reduction is presented in (**d**). The dashed line in (**b**,**d**) represents the linear fit and is shown for the orientation only.

The square wave potential steps explain how the linear actuation properties of the PPyCDC and PPyCDC-PT films changed in terms of the strain at the applied frequencies ranging from 0.0025 Hz to 0.1 Hz. Each chronopotentiogram calculated the charge density at each applied frequency, with the strain against the charge density, to analyze whether both film samples followed faradaic processes [48]. The strain against time for PPyCDC and PPyCDC-PT in the LiTFSI-PC electrolyte is presented in Figure 5a, with the strain against the charge densities at oxidation shown in Figure 5b. Those in LiTFSI-aq, with the strain–time profile (Figure 5c) and the strain against the charge density at reduction, are displayed in Figure 5d. The strain against the applied frequencies of both film samples in LiTFSI-PC is shown in Figure S1a, and the result for LiTFSI-aq is presented in Figure S1b.

The strain–time curves of the film samples in LiTFSI-PC (Figure 5a) revealed at the 0.0025 Hz frequency show a 2-times-higher strain for PPyCDC-PT (5.6%) in comparison to PPyCDC (2.9%). The PPyCDC films are comparable to PPy/DBS, with the CDC mostly located on the surface alone, which affects the elastic modulus (Table 1), being 2.8 times stiffer in comparison to PPyCDC-PT. The change in the elastic modulus of the PPy/DBS films after actuation has a direct effect on the strain [49]. Comparing the strain against the charge density at oxidation (Figure 5b), the strain increase for both film samples is nearly linear, revealing the faradaic nature [50] of the PPyCDC and PPyCDC-PT films. The charge density for PPyCDC and PPyCDC-PT is found in a similar range.

In the LiTFSI-aq electrolyte, the strain–time profile in Figure 5c showed a similar trend in comparison to Figure 5a, indicating that PPyCDC-PT had a 2.7-times-higher strain (6.8%) compared to the PPyCDC samples (2.5%). Another trend is observed in the strain profile, showing, in the case of PPyCDC, a slow increase after 100 s, nearly reaching a plateau (Figure 5c). In contrast, in the case of PPyCDC-PT, the strain increased continuously. The difference, we assume, is that in the case of PPyCDC-PT, due to the multi-charged CDC-PT^4-^, compensating for those with solvated Li^+^ ions at reduction, more time is required than that needed for the PPyCDC films. 

The strain against the charge densities at reduction in LiTFSI-PC and LiTFSI-aq is presented in Figure 5b and in Figure 5d, respectively. The strain increase with the increasing charge density is shown for the PPyCDC and PPyCDC-PT films. The strain for the PPyCDC-PT was, at all the charge densities in the LiTFSI-PC electrolyte (Figure 5b), higher than that of PPyCDC. The main reason for this relates firstly to the PPyCDC-PT elastic modulus of the films, which is much lower than that of the PPyCDC films (effect on the strain). Secondly, PPyCDC-PT has CDC-PT^4−^ particles incorporated in it, leading to more counterion incorporation at oxidation (anion-driven actuator) at deeper cavities, leading to better electron coupling. In the case of the LiTFSI-aq electrolyte (Figure 5d), we have cation-driven actuation with the much higher charged particles of CDC-PT^4−^, as well immobile DBS^-^ anions, leading to enhanced cation ingress and osmotic balance [51], and the PPyCDC-PT film swells faster in comparison to PPyCDC. In LiTFSI-aq (Figure 5d), the PPyCDC films had a 1.3-times-higher charge density at reduction than PPyCDC-PT. Due to the fact that the CDC particles are mostly located on the PPy surface (Figure 1b) and not covered with PPy, we assume that a non-faradaic process takes place. The EDX spectroscopy shown in Figure 3c indicates minor fluoride peaks for PPyCDC at oxidation and reduction. This implies that the cause is the positive charging of the CDC particles, the TFSI forming the EDL. At the same time, some of them tend to move into the nanopores of the CDC and stay in them [47], whereas at reduction, those remaining left negative charges balanced with the solvated Li^+^ cations in such a tendency, shown as well in the bending of the CDC actuators applied at different solvents [52].

In summary, the linear actuation properties of PPyCDC in PC or aq electrolytes are not affected by CDC particles, while the charge density was found to be slightly improved in the PPyCDC films. One reason for the lower strain values of the PPyCDC films is related to their higher elastic modulus than that of the PPyCDC-PT films. To investigate whether CDC in PPyCDC has an enhanced capacitance, chronopotentiometric measurements are described in the next section to determine the energy storage potential.

### 3.3. Energy Storage

Today, the demand for energy storage focuses on flexible capacitor materials [15]. In contrast, microporous CDC material (TiC-800 is applied in this research) with an average pore size distribution of 0.8 to 1 nm is of growing interest. The high surface area, which can be up to 1270 cm^2^g^−1^, makes CDC an ideal material for supercapacitors [14]. Therefore, our interest in this section focuses on the capacitor properties given by the combination of CDC with PPy/DBS regarding the PPyCDC and PPyCDC-PT films. The influence of the swelling of the film samples in either the PC or aq electrolyte is considered by calculating the volumetric capacitance *C_V_* using Equation (1):

(1) $$C_{V}=\frac{i}{-slope\cdot V}$$

**Figure 6:**
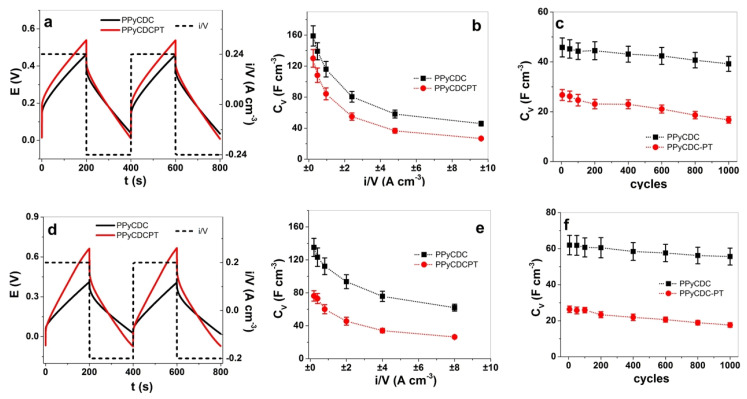
Chronpotentiometric measurements of PPyCDC (black line, ■) and PPyCDC-PT (red line, ●) with the variation in the frequency and current density using LiTFSI salt, with results showing the aqueous solvent potential time curve (3rd–4th cycle) with the current density (dashed line, ±0.24 A cm^−3^), presented in (**a**), the volumetric capacitance C_V_ against the applied current densities (from ±0.24 A cm^−3^ to ±9.6 A cm^−3^), presented in (**b**), and the long-term measurements of 1000 cycles (0.1 Hz, ±9.6 A cm^−3^), displayed in (**c**). The PPyCDC and PPyCDC-PT formed in the aqueous electrolyte, showing the potential time curve (±0.2 A cm^−3^) in (**d**), the volumetric capacitance against the applied current density in (**e**), and the long-term measurements at ±8 A cm^−1^ (1000 cycle, 0.1 Hz) in (**f**).

The slope is taken from the potential time curves at discharging (after IR drop) from each chronopotentiogram at the applied current density *i*/*V* (*i*: current, *V* = length ∙width ∙ thickness of film samples). The current densities (frequency range of 0.0025 Hz to 0.1 Hz) of the PPyCDC and PPyCDC-PT films in LiTFSI-PC ranged from ±0.24 A cm^−3^ to ±9.6 A cm^−3^ (constant charge density of ±48 C cm^−3^), and the films in the LiTFSI-aq electrolyte had current densities in the range from ±0.2 A cm^−3^ to 8 A cm^−3^, giving a constant charge of ±40 C cm^−3^. The potential time curves of PPyCDC and PPyCDC-PT in LiTFSI-PC at 0.0025 Hz are shown in Figure 6a. The volumetric capacitance against the applied current density is presented in Figure 6b. The volumetric capacitance retention (1000 cycles, at 0.1 Hz) at ±9.6 A cm^−3^ is shown in Figure 6c. The potential time profiles of the film samples investigated in the LiTFSI-aq electrolyte are displayed in Figure 6d, with the volumetric capacitance against the current densities in Figure 6e and the long-term measurements used to verify the capacitance retention shown in Figure 6f. For each film sample in each applied electrolyte, three identical samples were polymerized and measured, with the given mean values and standard deviations.

The potential time curves are shown PPyCDC and PPyCDC-PT in LiTFSI-PC in Figure 6a of, comparing the third and fourth cycles of each film sample. They are related to the ESCR model, defined under conditions with the charging/discharging in balance [45]. Several parameters can be read from the potential time curves, such as the maximum potential at oxidation and reduction, which was found to be enhanced by 1.2 times for PPyCDC-PT (PPyCDC, E_ox_: 0.46 V, E_red_: 0.04 V; PPyCDC-PT, E_ox_: 0.54 V, E_red_: 0.014 V). From the slope obtained from the discharging curves (Equation (1)), the volumetric capacitance at the different applied current densities shown in Figure 6b revealed, for the PPyCDC films, a 1.2-times-higher capacitance (159 ± 13 F cm^−3^) in comparison to PPyCDC-PT, with 130 ± 11 F cm^−3^ at the current density of ±0.24 A cm^−3^. In past research [53], conducting polymers showed a capacitance in an organic solvent in the range of 100 F cm^−3^, and our results fall in that range. Earlier research [54] discovered that smaller ions, at the doping of PPy deposited on latex in organic electrolytes, can have a capacitance of up to 300 F cm^−3^. It must be considered that conducting polymers such as PPy are pseudo-capacitors (redox reactive process). At the same time, CDC (EDL formation), a typical supercapacitor material shown in organic electrolytes [55], has a volumetric capacitance of up to 175 F cm^−3^. The capacitance in the long-term measurements (1000 cycles), shown in Figure 6c, revealed PPyCDC capacitance retention at 85%, while for PPyCDC-PT, 62% was found. 

The chronopotentiometry measurements of the LiTFSI-aq electrolyte, when comparing the potential time curves in Figure 6d, revealed differences in the possible evolution, showing, for PPyCDC-PT, a 1.6-times-higher voltage at oxidation of 0.66 V, and 0.41 V for PPyCDC. The discharge slope was much shallower compared to PPyCDC-PT, which directly reflects the volumetric capacitance. The CDC particles form a layer on the PPyCDC films, having a higher electrode surface area, affecting the capacitance (volumetric capacitance). At discharging (slope at discharging, Equation (1)), the non-faradaic process leads to faster discharging than the faradaic process (pseudo-capacitors) of the PPyCDC-PT films.

Figure 6e shows the best result for the PPyCDC in aqueous solvent in the range of 135 ± 11 F cm^−3^ at ±0.2 A cm^−3^. Similar values for PPy with porous carbon materials [4] were found in a comparable range of 107 F cm^−3^. In contrast, the charge/discharge time depreciation was the factor determining the achievement of a higher capacitance [56]. CDC materials have fast charging/discharging properties [57] based on the pore size. They are found to be in a particular range that ions can enter, being neither too small nor too large. 

In our case, EDX spectroscopy (Figure 3c) shows a certain amount of TFSI- in the charging/discharging state in an aqueous electrolyte. TFSI- is poorly solvated and has a van der Waals volume [58] of 144 Å^3^. Therefore, the pore size of the CDC materials, found to be 0.97 to 1.08 nm (TiC-800), applied in this work and identified in the previous research [59] can reach a volumetric capacitance of up to 80 F cm^−3^. The PPyCDC-PT films had a current density of ±0.2 A cm^−3^ and a nearly 1.8-times-lower capacitance of 76 ± 6 F cm^−3^ compared to the PPyCDC films. The capacitance retention of PPyCDC, shown in Figure 6f, was found after 1000 cycles to be in the range of 90%, and for PPyCDC-PT it was 67%, showing that PPyCDC, either in organic or aqueous electrolytes, has enhanced capacitance retention between 85 and 90%. Therefore, PPyCDC films are more suitable for energy storage devices, while PPyCDC-PT films are more pronounced in their linear actuation properties. 

## 4. Conclusions

The comparison of PPyCDC with CDC particles incorporated into the micelles in PPy doped with DBS^-^ films were compared to PPyCDC-PT films where CDC-PT^4−^ complexes were added to the PTA polyanions as additional dopants in electropolymerization. The differences between both the film samples, shown in the SEM images, indicate that PPyCDC has the most CDC particles on the surface of the films, which are mostly uncovered by PPy. Moreover, in the EDX spectroscopy, especially in the case of the LiTFSI-aq electrolyte, small amounts of fluoride were detected in the oxidation/reduction state, hinting that TFSI^-^ ions are incorporated into CDC pores. The PPyCDC-PT films revealed that all the CDC particles are surrounded by PPy, showing an enhanced strain that was 2 times higher in LiTFSI-PC and 2.7 times increased in the LiTFSI-aq electrolytes. We assume that the change in the elastic modulus of PPyCDC was found at 48 MPa after actuation, while PPyCDC-PT had 17 MPa (reduced by 2.8 times). Surprisingly, the charge density was 1.3 times higher for PPyCDC compared to PPyCDC-PT, which we assume is the effect of the CDC particles contributing to the charging/discharging capacitance. The energy storage properties favor the PPyCDC films, with a 1.2-times-higher volumetric capacitance in the organic electrolytes and 1.8-times-improved capacitance in the aqueous electrolytes, showing a higher retention capacitance after 1000 cycles (90%) compared to PPyCDC-PT, with 67%. The difference between the CDC with and without PTA can be differentiated, where PPyCDC-PT is favorable in terms of actuation, with an envisaged use in soft robotics. At the same time, PPyCDC should be applied in flexible energy storage devices. 

## Figures and Tables

**Table 1:** Electric conductivities σ_e_ and elastic modulus δ of PPyCDC and PPyCDC-PT films before and after actuation in the LiTFSI aq and PC electrolytes.

Film Samples	σ_e_ [S cm^−1^]			δ [MPa]	
	Pristine	LiTFSI-aq	LiTFSI-PC	LiTFSI-aq before (after)	LiTFSI-PC before (after)
PPyCDC	7.3 ± 0.5	5.2 ± 0.4	4.1 ± 0.3	52 ± 3.9 (48 ± 3.5)	31 ± 2.3 (87 ± 6.8)
PPyCDC-PT	9.8 ± 0.7	7.7 ± 0.6	4.8 ± 04	36 ± 2.1 (17 ± 1.2)	50 ± 4.1 (62 ± 4.8)

## Data Availability

The data presented in this study are available on request from the Corresponding author.

## Supplementary Materials

The following supporting information can be downloaded at: https://www.mdpi.com/article/10.3390/polym14214757/s1, Figure S1. Square wave potential steps of PPyCDC (■) and PPyCDC-PT (●) at applied at applied potential range 0.8 V to −0.5 V showing strain against frequencies (0.0025 Hz to 0.1 Hz) using LiTFSI salt in (a): propylene carbonate and (b): aqueous solvents.
