# Supplementary material for: Polypyrrole with Embedded Carbide-Derived Carbon with and without Phosphor Tungsten Acid: Linear Actuation and Energy Storage
Source: Polymers (Basel). 2022 Nov 6;14(21):4757. doi: 10.3390/polym14214757 (PMC9658178; doi:10.3390/polym14214757)
Supplement: Supplementary file 1 [file polymers-14-04757-s001.zip › polymers-1994894-supplementary.pdf]

Supplementary

Polypyrrole with Embedded Carbide-Derived Carbon with and without Phosphor Tungsten Acid: Linear Actuation and Energy Storage

Zane Zondaka [1], Quoc Bao Le [2] and Rudolf Kiefer [2,*]

[1] Intelligent Materials and Systems Lab, Institute of Technology, University of Tartu, Nooruse 1, 50411 Tartu, Estonia

[2] Conducting Polymers in Composites and Applications Research Group, Faculty of Applied Sciences, Ton Duc Thang University, Ho Chi Minh City 700000, Vietnam

* Correspondence: rudolf.kiefer@tdtu.edu.vn; Tel.: +84-784566419

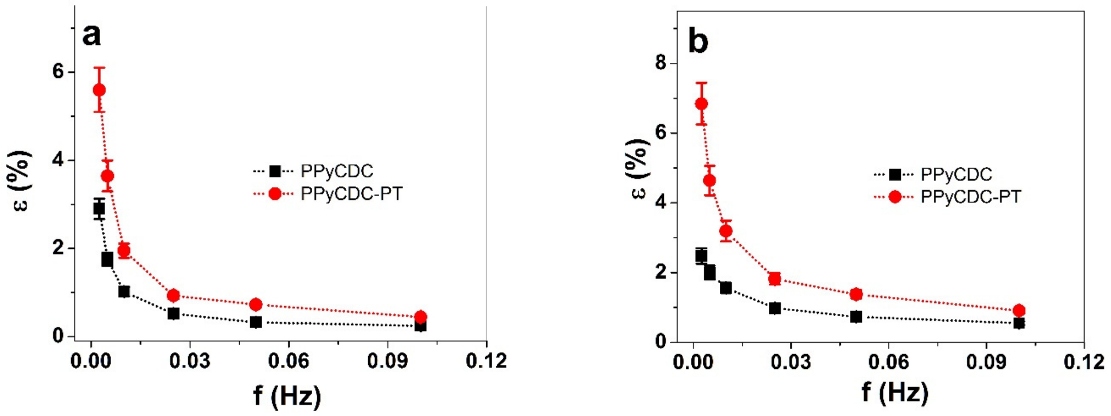

Figure S1. Square wave potential steps of PPyCDC (■) and PPyCDC-PT (●) at applied at applied potential range 0.8V to -0.5V showing strain against frequencies (0.0025Hz to 0.1Hz) using LiTFSI salt in a: propylene carbonate and b: aqueous solvents.
